# Low-Temperature Phosphine Fumigation Is Effective Against *Drosophila suzukii* in Sweet Cherry

**DOI:** 10.3390/insects16060635

**Published:** 2025-06-17

**Authors:** Hang Zou, Li Li, Jun Zhang, Baishu Li, Yu Xiao, Yonglin Ren, Ju Huang, Wei Chen, Tao Liu

**Affiliations:** 1 Chinese Academy of Quality and Inspection & Testing, Institute of Equipment Technology, Beijing 100123, China; zh1996nmg@126.com (H.Z.); liliice@126.com (L.L.); libaishu@163.com (B.L.); 2 Technology Innovation Center of Animal and Plant Product Quality, Safety and Control, State Administration for Market Regulation, Beijing 100123, China; 3 Chengdu Customs District, Chengdu 610041, China; 13568967531@163.com (J.Z.); cd19950176687@163.com (J.H.); 18584067627@163.com (W.C.); 4 College of Environmental and Life Science, Murdoch University, 90 South Street, Perth 6150, Australia; amy.xiao@murdoch.edu.au (Y.X.); y.ren@murdoch.edu.au (Y.R.)

**Keywords:** toxicity, large-scale verification, postharvest quality, efficacy

## Abstract

*Drosophila suzukii*, classified as a quarantine pest in some countries, poses a serious threat to global trade due to its ability to damage berries and cherries. Recent studies indicate that low-temperature phosphine (PH_3_) fumigation effectively controls this pest without compromising fresh produce quality. However, the specific protocol for managing *D. suzukii* larvae in cherries using this method remains underexplored. This study evaluates the efficacy of low-temperature PH_3_ fumigation in controlling *D. suzukii* larvae across three cherry varieties at 3 °C and investigates potential effects on key quality metrics. Tolerance assessments revealed that 3rd instar larvae exhibit the highest resistance to PH_3_ among all developmental stages. A concentration of 800 mL/m^3^ PH_3_ for 84 h at 3 °C achieved phytosanitary efficacy for 0.99997 with no negative effect on the quality attributes of the tested cherry varieties. These results support low-temperature PH_3_ fumigation as a viable postharvest treatment for *D. suzukii* management in sweet cherries.

## Simple Summary

*Drosophila suzukii* Matsumura (Diptera, *Drosophilidae*), a destructive invasive pest, poses a threat to global soft fruit production. This study evaluates low-temperature (3 °C) phosphine (PH_3_) fumigation as an eco-friendly alternative to chemical insecticides for controlling *D. suzukii* larvae in cherries. Results demonstrate high efficacy across all life stages of the pest in three cherry varieties, while maintaining fruit quality and shelf-life parameters. The treatment minimizes environmental impact and pesticide residues, addressing quarantine requirements and postharvest management challenges. By validating PH_3_ fumigation as a sustainable phytosanitary solution, this research advances practical, scalable pest control strategies for soft fruit industries, aligning with global demands for food safety and ecological preservation.

## 1. Introduction

Sweet cherry (*Prunus avium* (L.) Moench) belongs to the *Rosaceae*, plants of the genus Prunus, cherry. Native to the region bordering the Black Sea and the Caspian Sea in Eurasia, it has been cultivated for centuries [1,2]. Research indicates it was introduced to Europe from Turkey and later introduced to North America by immigrants. Cherries are prized by consumers for their sweet flavor, attractive appearance, and nutritional value, including anthocyanins, phenolic compounds, and vitamin C [3]. Global cherry production has grown steadily in recent decades, reaching 4.594 million tons in 2021 and 4.687 million tons in 2022. With approximately 266,700 hectares (ha) of cherry orchards nationwide—including 233,300 ha dedicated to sweet cherries—China now leads the world in sweet cherry cultivation area (https://www.mofcom.gov.cn/ (accessed on 1 Augest 2019)). Major production regions include Shandong Province and areas along the Longhai Railway. However, this fruit species is highly susceptible to pest infestations, particularly by *Drosophila suzukii* Matsumura (spotted wing drosophila, SWD), a quarantine pest classified under the *Drosophilidae* family (Diptera: Cyclorrhapha). This invasive fly poses a significant threat to cherry production in multiple countries, including the United States (USA), Canada, and European nations [4,5]. Therefore, phytosanitary treatment under quarantine conditions is required prior to export.

*D. suzukii* is native to Southeast and East Asia but has emerged as a destructive invasive pest in the USA and Europe [6,7]. Unlike most *Drosophila*, *D. suzukii* females possess serrated, rigid ovipositors that enable egg-laying directly into the epidermis of ripe, fresh fruit [8]. Larvae feeding within the fruit pulp induces softening, browning, and rotting, ultimately causing premature fruit drop and substantial yield losses [9,10]. Controlling *D. suzukii* is challenging due to its rapid reproduction, broad host range, and ability to infest a wide variety of thin-skinned, juicy fruits [11]. Over the past few years, researchers have explored different postharvest treatments for *D. suzukii*. Irradiation with a dose of 40 gray (Gy) can prevent 1st and 2nd instar larvae from developing into adults, while a dose of 80 Gy is necessary to prevent late pupae from emerging as adults that would produce the F1 generation of larvae [12]. Kim et al. (2016) further suggested that a higher dose of 150 Gy could induce adult infertility, aiding in the control of the spread of *D. suzukii* [13]. Chemical treatments, such as fumigation with ethyl formate at specific concentrations and temperatures, have also shown promise in controlling egg-stage *D. suzukii* without compromising blueberry quality [7]. Additionally, Yang et al. (2018) reported successful control of *D. suzukii* eggs and larvae using nitric oxide (NO) fumigation at different concentrations and temperatures [14]. Despite these advancements, the application of these methods often comes with drawbacks, including high costs, concerns over food safety, and potential negative impacts on fruit quality. Therefore, there is a pressing need to develop alternative treatment strategies that are both effective and safe.

Phosphine (PH_3_) has served as the primary fumigant for protecting stored bulk grains globally for decades, with its significance amplified in key applications following the phasing out of methyl bromide—a compound classified as ozone-depleting under the Montreal Protocol [15]. In recent years, the application of phosphine in different pest controls was conducted for exploration. Numerous reports show that PH_3_ fumigation at low temperature effectively controls various insect pests, such as the *Bactrocera tau* (at 5 °C) [16], *Bactrocera correcta* (at 5 °C) [17], *Frankliniella occidentalis* (at 3 °C; 5 °C) [18,19], and *Bactrocera dorsalis* (at 8 °C) [20]. But to also explore the effect of phosphine fumigation on fresh food quality, research has shown that low-temperature phosphine fumigation has no obvious phytotoxicity to lettuce (at 2 °C) [21], broccoli, asparagus, strawberry (at 2 °C) [22], white chrysanthemum (at 2 °C) [23], and the oriental lily (at 5 °C) [19]. However, adapting this methodology to control *D. suzukii* larvae in cherries requires careful consideration of various factors, including temperature, exposure time, and dose rates. Moreover, the effectiveness of this method against different stages of *D. suzukii* larvae and its potential impact on cherry quality need thorough investigation.

This study aims to evaluate the efficacy of low-temperature phosphine fumigation in controlling *D. suzukii* larvae at 3 °C in cherries, while also investigating its impact on cherry quality parameters. By conducting detailed experiments, we seek to identify an optimal fumigation schedule suitable for cherry exports. The insights gained from this study will contribute to the development of more effective and sustainable pest management strategies for cherry production, thereby fostering the development of specialized trade and ensuring the sustainability of agricultural endeavors.

## 2. Materials and Methods

### 2.1. Insect, Fumigant, and Fruit

Insect: A *D. suzukii* colony was obtained from the Institute of Plant Protection, Shandong Academy of Agricultural Sciences. This colony was originally collected from cherry, grape, and other fruit orchards of Shandong Province in 2022. Additionally, a wild population was collected for regular (<12 months) rejuvenation. *D. suzukii* were cultured on artificial diets. This culturing was performed under controlled laboratory conditions at 26 ± 2 °C with 75–85% relative humidity and a 12:12 h light–dark cycle at the Laboratory of Phytosanitary Treatment and Equipment, Chinese Academy of Inspection and Quarantine.

Fumigant: The ammonia-free PH_3_ gas was generated by reacting aluminum phosphide tablets (Nippon Kasei Co., Ltd., Tokyo, Japan) with a water solution, as previously described by Zou et al. (2024) [15].

Fruit: Cherry (country of origin: Shandong province) were purchased from the fruit market.

### 2.2. Developmental Duration Test

One hundred cherries were soaked in sterile water, washed, dried, and stored under laboratory conditions for 24 h to maintain temperature equilibrium. Then, they were put into the insect cage for natural infection for 12 h at room temperature, followed by incubation. From Day 1 (D1) to Day 11 (D11) after egg collection, ten cherries were dissected every 24 h to observe the developmental stages of *D. suzukii*. The experiment was conducted in three independent replicates.

### 2.3. Tolerance Comparison Test

Thirty cherry fruits were kept in *D. suzukii* rearing cages for 12 h. Each group of 6 fruits was subjected to rearing conditions for varying durations—12 h, 36 h, 60 h, 84 h, and 132 h—to obtain the various developmental stages of *D. suzukii*. The cherries were then placed in a 6 L fumigation chamber that had been chilled to 3 °C for 24 h beforehand. Subsequently, cherries infected with different developmental stages of *D. suzukii* were exposed to 100 mL/m^3^ of PH_3_ for 4 h at 3 °C.

Modified 6 L glass desiccators were utilized for the fumigation procedure. Each desiccators contained 6 infested cherries, with each fruit bearing 24–26 eggs (loading rate: roughly 0.8%). Desiccators were pre-equilibrated to 3 °C in an environmentally controlled chamber (KBF720, WTC Binder, Binder GmbH, Tuttlingen, Germany) for 24 h prior to gas exposure. After being hermetically sealed, precise amounts of PH_3_ gas were injected into the desiccators through Swagelok snap fittings (304, 3/8 in. i.d. × 8 mm, Shanghai Yihao Co., Shanghai, China), using a gas-tight syringe. For precision, measurements of the PH_3_ concentration were taken both 30 min post-injection and again 10 min prior to ventilation, employing a gas chromatograph (GC6890, Agilent Technology Co., Santa Clara, CA, USA) fitted with a thermal conductivity detector (TCD). Post-fumigation, the desiccators were opened for ventilation for 2 h, and subsequently, the cherries were maintained under optimal rearing conditions for differing durations.

For 12 h eggs, treated cherries were maintained for 72 h and incubated to the 2nd instar stage for survival statistics. 36 h eggs (1st instar larvae) were held for 48 h to reach the 3rd instar stage before analysis. The number of pupae was counted after the 60 h post-treatment eggs (2nd instar larvae) were kept at room temperature for 72 h. For 84 h eggs (3rd instar larvae), the number of pupae was counted after 48 h of incubation at room temperature. For the 132 h eggs (pupae), treated cherries were maintained for 120 h to record the number of adults. Larvae that did not respond when prodded with a blunt probe were considered dead. Mortality was calculated as the number of dead insects divided by the total number of insects. Because the total number of eggs at 12 h and 36 h was difficult to determine, the total number of 2nd instar larvae in control group was used for calculation.

### 2.4. Toxicity Assay

Based on the findings of the tolerance comparison test, cherries infested with the 3rd instar larvae of *D. suzukii* underwent fumigation with PH_3_ at a temperature of 3 °C. The fumigation, conducted for 4 h, was at varying concentrations of PH_3_, ranging from 50 to 1600 mL/m^3^, increasing by multiples of 2, to identify the optimal concentration for effectively controlling *D. suzukii* under low-temperature conditions. Following this, the infested fruits were treated with PH_3_ at the determined optimal concentration for durations from 0 h to 48 h at 3 °C, and the mortality rate of 3rd instar *D. suzukii* was recorded. For each test, 6 cherries, harboring approximately 130 larvae in total, underwent the specified fumigation procedure and then transferred to rearing conditions. Each treatment was replicated three times.

### 2.5. Large-Scale Test

After obtaining the results of the toxicity assay, the potential fumigation protocol underwent a validation test. In this experiment, 120 cherries were infested with *D. suzukii* in three rearing cages for 12 h. Subsequently, the cherries were kept in standard rearing conditions for 72 h to monitor the infestation levels. Additionally, 100 control cherries were randomly selected and maintained under the rearing conditions. Prior to evaluating the effectiveness of the fumigation, a larval count was conducted on both the treated and control cherries to accurately assess the fruit infestation rate [20].

A total of 660 cherries, infested with pests along with 152 healthy cherries intended for quality assessment, were placed into a 175 L stainless steel fumigation chamber with dimensions of 72 cm × 45 cm × 54 cm, which has been cooled to 3 °C for 24 h in a precisely controlled environmental chamber (KBF720, WTC Binder) to ensure optimal conditions for the fumigation process. During the fumigation process, the concentrations of PH_3_ and CO_2_ were measured 30 min following the initial injection and then periodically every 0.5 h, employing the identical methodology and equipment detailed earlier for PH_3_ analysis. When the fumigation was complete, the chambers underwent ventilation for 4 h in a fume hood. The infected cherries were kept under regular rearing conditions and underwent a thorough dissection 72 h later. Any larvae exhibiting movement or pupation were categorized as survivors. Concurrently, a control group of 76 untreated cherries was maintained at the fumigation temperature for 168 h.

### 2.6. Postharvest Quality Evaluation

Following the fumigation treatment, three varieties of cherry samples (Citrine, Red light, Mei zao) were randomly selected from both the treated and control groups at various intervals of 1 d, 7 d, and 14 d during storage at 3 °C. Samples were subjected to analyses to assess fruit appearance, respiration rate, relative conductivity, firmness, soluble solid content (SSC), and titratable acidity (TA). All experiments were conducted with three independent replicates.

#### 2.6.1. Visual Quality or Appearance

The appearances of cherries were recorded by a Canon camera (EOS 2000D, Canon Inc., Tokyo, Japan) after 14 days of treatment with low-temperature PH_3_ fumigation.

#### 2.6.2. Respiration Measurement

The respiration experiment adhered to a protocol published by Zou et al. [15]. Nine randomly selected samples of cherries from three different varieties (Citrine, Red light, Mei zao) were placed in 6 L glass containers. After incubating the samples at 0 h and after 4 h, a 0.4 mL gas sample was extracted. The concentration of CO_2_ in these samples was measured using gas chromatography (GC), and the respiration rates were calculated accordingly, expressed in milliliters per gram per hour (mL·g^−1^·h^−1^).

#### 2.6.3. Determination of Relative Conductivity

A double-edged blade was used to cut the peel of cherry fruit into a 1 mm thickness, with 1 g peel placed into a 50 mL beaker, followed by 20 mL distilled water. The solution was stirred at 25 °C for 20 min, and its electrical conductivity (D1) was measured with a DDS-307A (Rex Electric Chemical, Shanghai, China) conductivity meter. Then, the peel and the extract were boiled for 30 min. After cooling, distilled water was added to 25 mL, and the electrical conductivity (D2) was determined after mixing evenly. Relative conductivity was then calculated using the formula: Relative Conductivity = (D1/D2) × 100%. Five cherries were measured in each group and the experiment was repeated 3 times.

#### 2.6.4. Fruit Firmness, SSC and TA

For each treatment, 3 cherries were selected for firmness measurements using a TA-XT2i texture analyzer (Stable Micro Systems, Godalming, UK), following the methodology outlined by Cai et al. (2006) [24]. The probe, with a 5 mm diameter, was inserted 4 mm deep into the cherry flesh at a rate of 1 mm/s.

Following the firmness assessments, juice samples were extracted from the same 3 cherries used earlier. The soluble solid content (SSC) was determined using a handheld refractometer (GMK-701R, G-won Hitech Co., Ltd., Seoul, Republic of Korea), while the titratable acidity (TA) was measured with an acidity meter (GMK-855, G-won Hitech Co., Ltd., Republic of Korea).

### 2.7. Data Analysis

Mortality rates were corrected using Abbott’s formula. The means and standard error (SE) of three replicates were arranged and categorized using Excel (2021) statistical software. SPSS 16.0 software from IBM Corporation was employed to identify differences between the tolerance test and the toxicity test treatment group and the control group. The Tukey HSD test analysis was used to compare treatment means at a significance level of *p* < 0.05. Probit analysis was performed using Polo Plus software (Version: 2.0), using time as the independent variable and the mortality probability value as the dependent variable (LeOra Software, Berkeley, CA, USA). The estimated efficacy (1 − Pu), was determined using a designated formula,

(1) $$1-Pu={\left(1-C\right)}^{1/n}$$

where C represents the level of confidence and *n* denotes the quantity of test insects involved [25].

## 3. Results

### 3.1. Development of D. suzukii in Cherry Fruit

**Figure 1:**
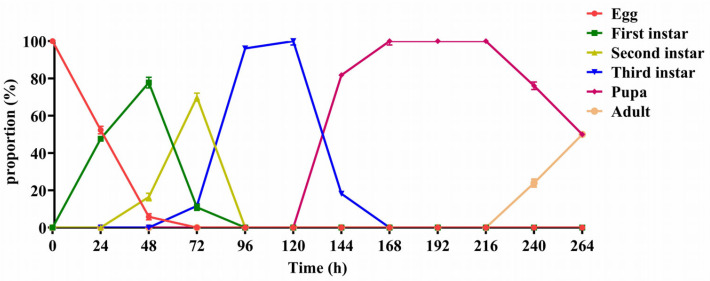
Proportions of various developmental stages (egg, first instar larva, second instar larva, third instar larva, pupa and adult) of *D. suzukii* in infested cherry incubated at 26 ± 2 °C for 0–264 h. Data are expressed as means ± SE.

The proportion of each developmental stage is shown in Figure 1 for *D. suzukii* cultured in naturally infected cherry for 24 h to 264 h. Eggs began to hatch after 24 h, and by 48 h, most of the 1st instar larvae were observed, with a few having developed into the 2nd instar. By 72 h, about 70% of the larvae had reached the 2nd instar stage. From 96 h to 120 h, the larvae were mainly in their 3rd instar, while from 144 h to 216 h, they were concentrated in the pupal stage. At 240 h, the first adults emerged, and by 264 h, 50% of the pupae had emerged as adults.

### 3.2. Third Instar Larvae Exhibit Highest Tolerance to PH_3_ Fumigation in D. suzukii

The survival rates of *D. suzukii* at different developmental stages under phosphine fumigation at 3 °C are shown in Table 1. Egg stage and pupal stage were the most sensitive to phosphine fumigation, followed by second and 1st instar larvae. The results showed that the 3rd instar *D. suzukii* larvae in cherry fruits had the strongest tolerance. These larvae were used for the subsequent toxicity test.

### 3.3. Toxicity Test-Screening for Optimal Phosphine Treatment Concentration

Phosphine treatment with different concentrations at 3 °C was used to determine the optimal fumigation concentration. In the range of 0 mL/m^3^ to 800 mL/m^3^, the mortality rate of the 3rd instar larvae of *D. suzukii* increased with the increase in concentration. However, compared to 800 mL/m^3^, there was no significant difference in mortality at 1600 mL/m^3^, indicating that 800 mL/m^3^ is the optimal fumigation concentration of phosphine (Table 2).

The phosphine concentration of 800 mL/m^3^, which can effectively control the 3rd instar larvae of *D. suzukii*, was used for treatment. The probit analysis results showed that the heterogeneity value of the probit model was small (<1), indicating a good fit between estimation and data. Based on the resulting probit 9 value of 90.979 h (95% CI:72.38~118.44), 84 h was selected as a potential treatment schedule for further validation tests (Table 3).

According to statistics, the infection rate of 3rd instar *D. suzukii* was 21–27 larvae/cherry. As shown in Table 4, 31,186, 30,166, and 30,635 larvae from different varieties of cherry were treated according to the infection rate; no survivors were found, and the treatment efficiency reached 0.9997.

### 3.4. Quality Evaluation of Cherries

#### 3.4.1. Visual Quality

**Figure 2:**
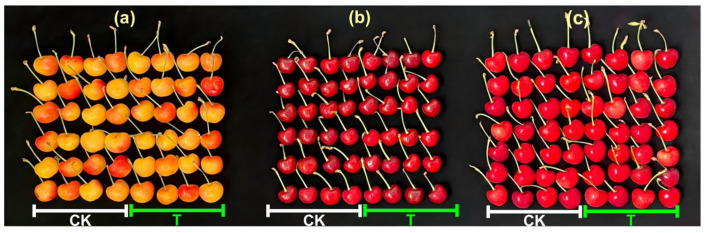
Effects of 800 mL/m^3^ PH_3_ fumigation for 84 h at 3 °C on the appearance of three cherry varieties: (**a**) Citrine, (**b**) Red light, (**c**) Mei zao. CK: control group; T: treatment group.

Based on the results of toxicity test, we evaluated the effects of PH_3_ treatment at 3 °C for 84 h on the appearance quality of three cherry cultivars. As shown in Figure 2, there was no visible difference in the appearance of the three types of cherries 14 days after fumigation, compared to the control group.

#### 3.4.2. Firmness

**Figure 3:**
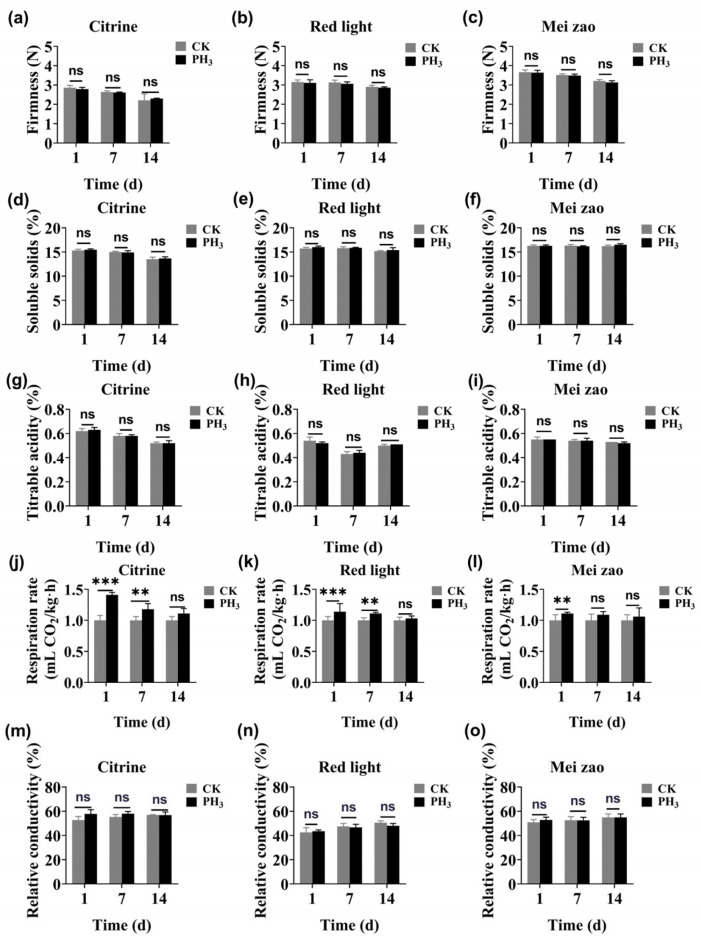
(**a**–**c**) Firmness, (**d**–**f**) soluble solids, (**g**–**i**) titrable acidity, (**j**–**l**) respiration rate, and (**m**–**o**) relative conductivity of Citrine, Red light, and Mei zao after fumigation with 800 mL/m^3^ PH_3_ for 84 h at 3 °C. Data are presented as mean ± SE (standard error), with significant differences denoted by different letters based on a Tukey HSD test at *p* < 0.05 (ns: *p* > 0.05, ** *p* < 0.01, *** *p* < 0.001). CK represents the control cherries.

As can be seen from Figure 3a–c, there are differences in the hardness of cherries among different varieties, with the Citrine varieties being softer than that of the Mei zao and Red light varieties. However, the hardness test results indicated that the hardness of the three kinds of cherries remained unchanged after fumigation.

#### 3.4.3. Soluble Solids

Figure 3d–f shows that the changes in the soluble solids content of three cherry varieties after fumigation. The levels of soluble solids in the three varieties were basically consistent, and the fumigation treatment did not have a negative effect on them.

#### 3.4.4. Titrable Acidity

By measuring acidity, it can be observed that Citrine varieties exhibited the highest-level titrable acidity, followed by Mei zao. Furthermore, there was no significant difference in the titrable acidity of the three cherry varieties after 14 days of fumigation compared to the control (Figure 3g–i).

#### 3.4.5. Respiration Rate

As shown in Figure 3g–l, the change in respiration rate is more obvious than other indicators. The Citrine varieties and Red light varieties showed a significant increase in respiration rate during the first day and again on the seventh day. In contrast, the control group’s respiration rate remained relatively stable. After treatment, the respiration rate of Citrine varieties increased but returned to normal levels by the 14th day. The Meizao varieties also showed a significant increase in respiration rate on the first day, but it had recovered to normal levels by the seventh day.

#### 3.4.6. Relative Conductivity

In Figure 3m–o, the conductivity of the three kinds of cherries, when fumigated, has not changed significantly.

## 4. Discussion

Phosphine (PH_3_), widely used as a fumigant for pest control in stored grains and commodities, is particularly effective when combined with carbon dioxide, decomposing into harmless phosphates [26,27,28]. Its advantages encompass effective pest penetration, absence of residue, and low cost, despite the consideration that long-term exposure may pose a drawback [9,29,30]. Low temperatures, primarily utilized for disinfecting stored agricultural products from pests or for quarantine purposes, have also demonstrated effectiveness against pests such as *Ceratitis capitata* and *Anastrepha suspensa* [31,32]. Furthermore, they can significantly reduce adult emergence in fruits like blueberries and strawberries, thereby extending their shelf life [33]. Thus, low temperatures present a viable option not only for pest control but also for maintaining the marketability of fruits [33,34]. The objective of this research was to identify the most tolerant life stage of *D. suzukii* to phosphine fumigation under low-temperature conditions; to determine mortality trends across various phosphine concentrations; to evaluate the impact of treatment conditions on the quality of cherry fruits; and to establish a safe and practical fumigation protocol. This research is critical to managing the economically crucial fruit fly pest and provides a fresh perspective and scientific rationale for developing innovative pest control strategies.

In examining *D. suzukii* tolerance to low-temperature phosphine fumigation across developmental stages, we observed a phenomenon running counter to common perceptions. While the pupal stage is generally regarded as the most tolerant insect stage to fumigants, our findings indicate that the final instar larval stage exhibits the highest tolerance to low-temperature phosphine treatment (Table 1). This result contrasts with previous studies, including work by S. Manivannan (2015), who found that the pupal stage of *Tribolium castaneum* exhibited the greatest resistance to low-temperature phosphine fumigation [35]. However, our findings are more consistent with those of Ma et al. (2024), who observed that among the different developmental stages of *B. dorsalis*, the 3 L stage shows a higher tolerance to phosphine [36], which may be related to specific physiological mechanisms and metabolic pathways in the larvae. In view of this phenomenon, we think that the late-stage larvae may accumulate certain stress resistant substances or mechanisms during development, so that they can resist the toxic effects of low-temperature phosphine. This echoes the findings of C. Austin et al. (2019), who found that the late larval stage of *Drosophila melanogaster* is more adaptive to temperature changes than the pupal stage [37]; similarly, Kathrine E. Pedersen et al. (2020) also observed that the final larval stage of the beetle *Tenebrio molitor* is more tolerant to insecticide than the pupal stage due to differences in uptake, elimination, and detoxifying enzyme activity [38].

When examining the relationship between phosphine concentration and *D. suzukii* mortality, we observed a dose-dependent increase in mortality across the 200–800 mL/m^3^ range, but the mortality plateaued at 1600 mL/m^3^ (Table 2). This finding is similar to some previous research; for example, Evagelia Lampiri (2021) found that high concentrations of phosphine can have a paralyzing effect on insects, and there exists a “sweet spot” where mortality does not increases with increasing concentration [39]. Additionally, the influence of the insect supercooling point effect cannot be ruled out. According to J. Mckenzie et al., the supercooling point temperature of *D. melanogaster* is around 13 °C [40]. Meanwhile, Štětina et al. (2019) reported that supercooling triggers the immune response in *Drosophila* larvae that enhances their stress resistance [41]. Therefore, it is speculated that phosphine treatment at 3 °C might cause the internal temperature of insects to plummet, triggering the mechanism of the supercooling point, and thereby improving their tolerance to fumigants. However, this situation may need to be considered by examining the interaction between the cold tolerance mechanism of insects and the toxicity of phosphine. In order to more accurately understand this phenomenon, further studies will be carried out to explore the physiological response and adaptive mechanism of insects under low temperature stress and phosphine treatment.

To further explore and refine control methods for *D. suzukii*, we carried out systematic experiments and analyses. By evaluating the toxicity of phosphine at different developmental stages of the insect and applying probit analysis, we established treatment indexes for effectively managing *D. suzukii* (Table 3). The indexes, which take into account specific phosphine concentrations and precise temperature conditions, provide a crucial basis for developing a practical and effective fumigation protocol. Our approach is similar to that of Zou et al. (2024) [15], who established an experimental system to assess the impact of fumigants on pest control. Furthermore, we also assessed the impact of the treatment conditions on cherry fruit quality. By examining key quality indicators, including appearance, firmness, acidity, and electrical conductivity, we confirmed that low-temperature phosphine treatment effectively controls *D. suzukii* without compromising cherry fruit quality (Figure 2 and Figure 3). These results align with Liu’s (2008) findings that low-temperature phosphine fumigation efficiently controls pests of perishable commodities without compromising berry quality [42]. However, caution is warranted in interpreting these results, as fumigation efficacy may vary considerably depending on ecological factors including fruit species, maturity stage, and the presence of biotic competitors or natural enemies.

In summary, this study identified the late larval stage as the most tolerant instar during low-temperature phosphine fumigation for controlling *D. suzukii*, as well as examined mortality trends and potential underlying causes across various phosphine concentrations. These findings not only provide an interesting contrast and complement to previous research but also serve as a crucial reference for the fruit industry to develop more sustainable and effective pest management strategies. Future research should focus on refining this fumigation technique and exploring its applicability in other crops and geographical regions. Additionally, in order to fully understand the response mechanism of insects to fumigants, it is necessary to deepen the exploration and research into their stress resistance mechanism and physiological adaptability.

## Figures and Tables

**Table 1:** Survival rate of different life cycles of *D. suzukii* following a 4 h exposure to 400 mL/m^3^ PH_3_ at 3 °C.

Instar	4 h			
	No. of Insects	Mortality ± SE (%)	No. of Insects	Corrected Mortality ± SE (%)
Egg	436	10.43% a	533	90.77% a
1st instar Larva (1 L)	329	8.31% ab	372	62.41% b
2nd instar Larva (2 L)	322	6.53% ab	357	71.36% b
3rd instar Larva (3 L)	359	1.69% b	278	20.09% c
Pupa	130	1.70% b	520	90.19% a
ANOVA	F = 6.83; df = 4, 10; *p* < 0.01		F = 181.22; df = 4, 10; *p* < 0.001	

Distinct letter denote statistical variations in the mean survival across various insect stages (*p* < 0.05). SE, standard error.

**Table 2:** Mortality of 3rd instar larvae of *D. suzukii* exposed to different concentrations of PH_3_ at 3 °C for 4 h.

PH_3_ (mL/m^3^)	4 h		
	No. of Insects	Mortality ± SE (%)	Corrected Mortality ± SE (%)
0	160	0.61 ± 0.86 d	0.00 ± 0.00 f
50	419	23.16 ± 3.08 e	22.70 ± 2.67 e
100	318	33.61 ± 4.03 d	31.75 ± 1.95 d
200	195	41.90 ± 1.47 c	41.71 ± 1.47 c
400	234	72.56 ± 4.52 b	73.22 ± 4.3 b
800	267	82.70 ± 1.93 a	82.58 ± 2.1 a
1600	182	85.83 ± 2.84 a	85.72 ± 2.97 a
ANOVA		F = 243.16; df = 6, 14; *p* < 0.001	F = 339.08; df = 6, 14; *p* < 0.001

Distinct letter denote statistical variations in the mean mortality across various insect stages (*p* < 0.05). SE, standard error.

**Table 3:** Probit analysis of 3rd instar larvae of *D. suzukii* treated with 800 mL/m^3^ PH_3_ at 3 °C.

Treatment	*n*	Slope ± SE	Hetero. ^a^	LT_50_ (95% CI) (Lower–Upper)	LT_90_ (95% CI) (Lower–Upper)	LT_99_ (95% CI) (Lower–Upper)	LT_99.9968_ (95% CI) (Lower–Upper)
Low Temperature PH_3_ fumigation	2732	2.513 ± 0.097	0.94	2.33 (2.11~2.56)	10.51 (9.49~11.77)	19.63 (17.18~22.29)	90.98 (72.38~118.44)

^a^ Heterogeneity.

**Table 4:** Efficacy analysis of a large-scale test of *D. suzukii* on three cherry varieties (Citrine, Red light, Mei zao).

Time	Temperature	Concentration of PH_3_	Variety of Cherry	Number of Larvae	Phytosanitory Treatment Efficacy
84 h	3 °C	800 mL/m^3^	Citrine	31,186	0.9997
			Red light	30,166	0.9997
			Mei zao	30,635	0.9997

## Data Availability

The original contributions presented in this study are included in the article. Further inquiries can be directed to the corresponding author.
